# Canine Distemper Outbreaks in Wild Carnivores in Northern Italy

**DOI:** 10.3390/v13010099

**Published:** 2021-01-13

**Authors:** Tiziana Trogu, Sabrina Canziani, Sara Salvato, Alessandro Bianchi, Irene Bertoletti, Lucia Rita Gibelli, Giovanni Loris Alborali, Ilaria Barbieri, Alessandra Gaffuri, Giovanni Sala, Enrica Sozzi, Davide Lelli, Antonio Lavazza, Ana Moreno

**Affiliations:** Istituto Zooprofilattico Sperimentale della Lombardia e dell’Emilia Romagna “Bruno Ubertini” (IZSLER), Via Antonio Bianchi 7/9, 25124 Brescia, Italy; sabrina.canziani@izsler.it (S.C.); sara.salvato@izsler.it (S.S.); alessandro.bianchi@izsler.it (A.B.); irene.bertoletti@izsler.it (I.B.); luciarita.gibelli@izsler.it (L.R.G.); giovanni.alborali@izsler.it (G.L.A.); ilaria.barbieri@izsler.it (I.B.); alessandra.gaffuri@izsler.it (A.G.); giovanni.sala@izsler.it (G.S.); enrica.sozzi@izsler.it (E.S.); davide.lelli@izsler.it (D.L.); antonio.lavazza@izsler.it (A.L.); anamaria.morenomartin@izsler.it (A.M.)

**Keywords:** Alps, Italy, wildlife, canine distemper, phylogenetic analysis

## Abstract

Canine distemper (CD) is a fatal, highly contagious disease of wild and domestic carnivores. In the Alpine territory, several outbreaks have occurred in the past few decades within wild populations. This study investigated the presence of canine distemper virus (CDV) infections in wild carnivores in Lombardy, relating to the different circulating genotypes. From 2018 to 2020, foxes, badgers, and martens collected during passive surveillance were subjected to necropsy and histological examination, showing classical signs and microscopic lesions related to CDV. Pools of viscera from each animal were analysed by molecular methods and immunoelectron microscopy. Total prevalences of 39.7%, 52.6%, and 14.3% were recorded in foxes, badgers, and stone martens, respectively. A phylogenetic analysis showed that the sequences obtained belonged to the European 1 lineage and were divided into two different clades (*a* and *b*) according to the geographical conformation of alpine valleys included in the study. Clade *a* was related to the European outbreaks originating from Germany in 2006–2010, while clade *b* was closely related to the CDV sequences originating from northeastern Italy during the 2011–2018 epidemic wave. Our results suggest that CDV is currently well adapted to wild carnivores, mostly circulating with subclinical manifestations and without severe impact on the dynamics of these populations.

## 1. Introduction

Canine distemper virus (CDV) is the causative agent of a fatal disease in wild and domestic carnivores. It is an enveloped, negative-sense, single-stranded RNA virus belonging to the family *Paramyxoviridae*, genus *Morbillivirus*. Its genome encodes for six structural proteins; in particular, hemagglutinin (H) is an integral membrane glycoprotein involved in the attachment to cell receptors in the first step of the infection, promoting the fusion of membranes. The heterogeneity within the H genes allows the distinction of at least nine CDV lineages mainly according to the geographical distribution pattern: America 1 (NA1), America 2 (NA2), Europe 1/South America 1 (EU1/SA1), Europe 2/Europe-wildlife (EU2), Europe 3/Arctic-like (EU3), Asia 1 (AS1), Asia 2 (AS2), South Africa (ZA), and South America 2 (SA2) [1].

Canine distemper (CD) is a highly contagious disease characterised by a high morbidity and mortality. Virus transmission is known to occur mainly through aerosols and contact with respiratory and ocular fluids and exudates, although other body excretions and secretions (e.g., urine and faeces) could contribute to viral shedding during the acute phase of infection [2]. Trans-placental infection has also been documented, at least in domestic dogs [3].

The infection severity depends on the immune status and age of the host species and the virulence of the strain. It is estimated that 50–70% of CDV infections in canids are subclinical and characterised by non-specific symptoms or a mild self-limiting respiratory disease. Clinical manifestations are mostly observed in immune-compromised animals, recognising two main clinical forms: an acute systemic form and a chronic nervous form characterised by abnormal behaviour, incoordination, and convulsions up to paralysis [4].

CDV can affect a very broad range of host species worldwide. Canidae and Mustelidae families are the most affected, but CDV has also been detected in the Felidae, Viverridae, Procyonidae, and Ursidae families [2,5]. Natural cases of CDV have been documented in a Japanese macaque (*Macaca fuscata*) [6] and collared peccaries (*Pecari tajacu*) [7]. Moreover, CDV infection was recognised as the primary cause of death in thousands of seals (*Pusa caspica*) in the Caspian Sea in 2000 [8]. Indeed, several CDV-related morbilliviruses have been detected in marine mammals, causing severe diseases. This is likely due to the strong aggregation and social behaviour of these populations living at the marine–terrestrial interface, which allowed the potential transmission of CDV, which is usually recorded in terrestrial carnivores.

Pertaining to the occurrence of CDV among wild species in Italy, in the last decade several outbreaks have occurred in foxes (*Vulpes vulpes*), badgers (*Meles meles*), and stone martens (*Martes foina*) in the Alpine regions of northeastern Italy, particularly the Trentino-Alto-Adige, Veneto, and Friuli Venezia Giulia regions [9]. The virus easily disseminated, quickly reaching the Italian Pre-Alps and urbanised areas [10], South Bavaria [11], and Switzerland [12]. At the same time in Southern Italy in 2013, CDV was identified mainly in wolves, but it was also found in foxes and badgers [13]. Phylogenetic analyses carried out during these outbreaks highlight the presence of three lineages with a defined geographical distribution pattern. In particular, two different lineages, EU1 and EU2, were detected in Northern Italy [13], and they seemed to have originated commonly from the Eastern Europe outbreaks. Indeed, they clustered together with viruses isolated from Hungarian dogs in 2004 [10]. Conversely, in the southern part of the country the EU3 lineage was predominant [14]. This virus, identified for the first time mainly in wolves [13], was the same strain as that previously recognised in domestic dogs, thus suggesting virus transmission from dogs to wolves. Thus, infected domestic dogs are likely the primary source of EU3 lineage for wildlife [14], and, at least in Italy, both the uncontrolled trading of low-cost pets from Eastern Europe and the presence of feral dogs probably represents the cause of CDV circulation among wild carnivores [13]. However, these viruses seem to circulate in wild animals with extremely low or asynchronous infection levels compared with those in domestic dogs, suggesting CDV persistence in other wildlife species within complex reservoir systems [15].

In an alpine environment, recurrent infections occur in wild carnivores. Moreover, recent studies carried out in Switzerland in 2009 have highlighted the circulation of strains similar to the Snyder–Hill-like strain [16], with accentuated neurotropism and characterised by high morbidity and mortality [12]. Since the beginning of 2018, there has been an increase in mortality in foxes infected with CDV in northeastern Italy, as highlighted through passive wildlife surveillance. In June 2018, CDV was also detected southward in the pre-alpine and alpine territories of the Lombardy region in some foxes and subsequently in badgers and stone martens. Considering the potential impact of CDV on wildlife population dynamics, especially endangered species, we aimed to analyse wild carnivores which were found dead and regularly collected under the framework of the regional wildlife monitoring and surveillance plan. Since animals could be found several days after the precise time of death with evident post-mortem alterations or with altered morphology due to the traumatic impacts of collision with vehicles or predation, in such cases the signs and lesions of CD could disappear, leading to the exclusion of CDV as the cause of death. Thus, considering the new re-emergence of CDV, this study aimed to systematically investigate and quantify the presence of CDV infections in wild carnivores in Lombardy and characterise the different circulating genotypes in order to better understand the virus origin and distribution among wild animal populations better.

## 2. Materials and Methods

### 2.1. Sampling

**Figure 1:**
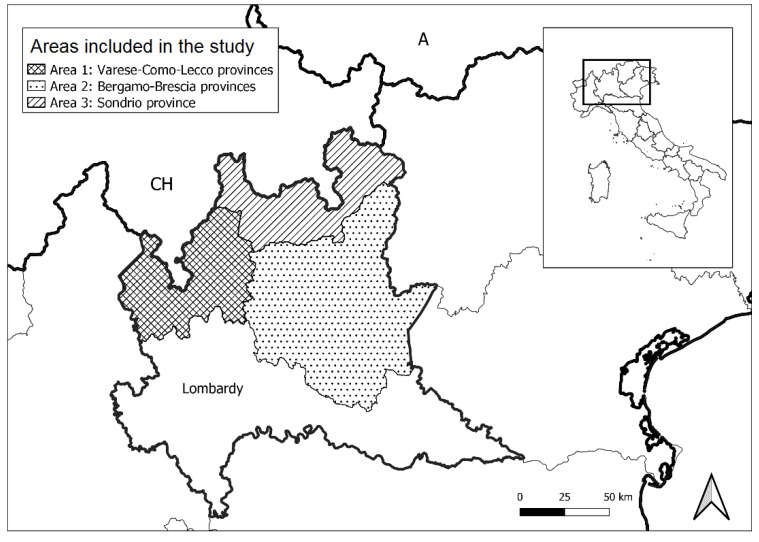
Representation of the three areas within the Lombardy region in north Italy.

From June 2018 to February 2020, a virological survey about the presence of CDV in wild carnivores was carried out in Northern Italy. Particularly, in the Lombardy region, carcasses of wild animals obtained from passive surveillance were conferred to the diagnostic laboratories of “Istituto Zooprofilattico Sperimentale della Lombardia e dell’Emilia Romagna.”(Brescia, Italy). Overall, samples from 126 foxes (*Vulpes vulpes*), 19 badgers (*Meles meles*), and 7 stone martens (*Martes foina*) were collected and subjected to necropsy and histological examination. Most of the subjects were found in the northern part of the region, characterised by pre-alpine and alpine environments. In particular, three areas constituted by contiguous territories were identified (Figure 1). Area 1 represents the northwestern region (Varese, Como, and Lecco provinces); area 2 corresponds to the central region (Bergamo and Brescia provinces), and area 3 represents the northern region of Lombardy (Sondrio Province) close to the border with Switzerland. Areas 1 and 2 are characterised by the presence of plains in the southern part, wide lacustrine environment, and the presence of mountains in the northern part. Area 3 is mostly characterised by an alpine environment with a wide central valley which connects Valchiavenna and High Valtellina, two important routes to Switzerland.

In particular, we collected 41 foxes, 3 badgers, and 1 stone marten in Area 1; 20 foxes and 2 badgers in Area 2; and 65 foxes, 14 badgers, and 6 stone martens in Area 3. Information about wild carnivores’ distribution in Italy is limited. Currently, no population estimates are available in the considered areas for any of the target species.

### 2.2. Anatomo-Pathological and Histopathological Investigations

Full necroscopic examinations were performed only when the post-mortem alterations were not so advanced as to prevent it. Independently from that, pools of tissue specimens from the brain, lungs, stomach, intestine, and bladder were systematically taken during the post-mortem investigation. Suitable samples were also submitted to histopathology. The samples were fixed in 10% buffered formalin, processed, sectioned at 5 μm in thickness, and stained with Hematoxylin and Eosin (HE) according to standard protocols.

### 2.3. Sequencing and Phylogenetic Analyses

A portion of each sample was diluted 1:10 in phosphate-buffered saline containing 1% penicillin and streptomycin and 10% glycerol and then homogenised. After centrifugation at 3750 rpm for 15 min, viral RNA was extracted from 250 µL of supernatant using the QIAsymphony™ SP Instrument (Qiagen, Hilden, Germany) according to the manufacturer’s instructions. Negative and positive controls were included in the extraction.

First, a screening PCR was performed for diagnostic purposes on the highly conserved nucleoprotein gene, which represents a suitable target for the detection of all CDV strains [17]. A fragment of 287 bp was amplified by RT-PCR using the commercial Qiagen One Step RT-PCR kit (Qiagen). PCR was performed with a total reaction volume of 25 µL, containing 5 µL of extracted RNA, 5 µL of 5× Buffer, 5 µL of 10 mM dNTPs, 1 µL of MIX enzyme, 1 µL of 40 U/µL RNAse inhibitor, 1 µL of RNAse-free water, and 1 µL each of 20 µM CVD P1 forward (5′-ACAGGATTGCTGAGGACCTAT-3′) and CDV P2 reverse (5′-CAAGATAACCATGTACGGTGC-3′) primers [18]. After reverse transcription, cDNA was denatured at 95 °C for 15 min, and the reaction comprised 45 cycles of a denaturation step at 94 °C for 1 min, an annealing step at 59.5 °C for 2 min, an extension step at 72 °C for 1 min, and a final extension step at 72 °C for 10 min.

Secondly, the partial sequencing of the H gene (484 bp) was performed on the positive samples from the first PCR to identify the different genotypes circulating during the outbreak. We used specific forward (5′-CTTGCTTGCTATCACTGGAG-3′) and reverse (5′-TTTTGAAATCAAAGACATGG-3′) primers [12]. PCR was performed using SuperScript™ III Reverse Transcriptase (Invitrogen Corporation, Carlsbad, CA, USA) with a 25 µL total reaction volume, which contained 5 µL of extracted RNA, 1 µL each of 0.15 µM primers, 12.5 µL 2× Reaction Mix, 1 µL of Superscript III, and RNAse-free water. The thermal cycle was performed for initial reverse transcription, followed by denaturation at 94 °C for 2 min and 40 cycles of a denaturation step at 94 °C for 15 s, an annealing step at 51 °C for 30 s, an extension step at 68 °C for 1 min, and a final extension step at 68 °C for 5 min. Amplicons from both the PCRs were analysed by electrophoresis in 2% agarose gel stained with Eurosafe Nucleic acid Stain (Thermo Fisher Scientific, Waltham, MA, USA). Partial H gene sequences were compared with sequences of the reference and field CDV strains originating from different countries and wild and domestic animal species available in GenBank. A phylogenetic tree was constructed using the maximum likelihood method in the IQ-tree software [19] with bootstrap analyses involving 1000 replicates. The best-fit model HKY + F + G4, identified by ModelFinder, was applied. Based on the phylogenetic data, the complete H gene was amplified in 9 Italian strain representatives of the two observed groups originating from 5 foxes, 3 badgers, and 1 stone marten (Table 1). Finally, four different primer pairs were used, and RT-PCR was conducted using a one-step RT-PCR kit (Qiagen), as previously described [11]. All the PCRs were conducted under the same conditions using a 25 µL reaction volume containing 5 µL of extracted RNA and 1 µL each of 0.6 µM primers. The thermal cycle included heating at 50 °C for 30 min, followed by denaturation at 95 °C for 15 min and 45 cycles of a denaturation step at 94 °C for 30 s, an annealing step at 50 °C for 1 min, an extension step at 72 °C for 1 min, and final extension at 70 °C for 10 min. Sequences were aligned with other CDV hemagglutinin protein sequences from GenBank using the Lasergene sequencing analysis software package (DNASTAR, Madison, WI, USA), and a phylogenetic analysis was performed according to the modality mentioned earlier. Potential N-linked glycosylation sites were predicted using the NetNGlyc server for the prediction of amino acid sequences (http://www.cbs.dtu.dk/services/NetNGlyc/). Only asparagine (Asn) residues that occur within the Asn-Xaa-Ser/Thr triplet with a threshold value of 0.5 were considered.

### 2.4. Immunoelectron Microscopy (IEM)

Negative staining immunoelectron microscopy (IEM) was performed as previously described [20]. Briefly, the remaining portions of the tissue/organ samples were diluted 1:5 *w/v* in distilled water and mechanically homogenised. Then, 100 μL of the supernatants obtained after double centrifugation at 6000 and 10.000 rpm for 30 min, to eliminate gross debris, were incubated for 1 h at 37 °C with gentle agitation along with an equal amount of a pool of sera taken from hyperimmunised vaccinated dogs containing high levels of antibodies against CDV. Thereafter, the samples were ultra-centrifuged in Airfuge Beckman (Beckman Coulter Inc. Life Sciences, Indianapolis, IN, USA) at 21 psi for 15 min and subjected to negative staining using phosphotungstic acid (NaPt) (2%, pH 6.8) for 90 s. Samples were observed using a FEI Tecnai G2 Spirit Biotwin transmission electron microscope (Thermo-Fischer/FEI, Eindhoven, The Netherlands) operating at 85 kV and 19,000–43,000×.

### 2.5. Statistical Analyses

Data were analysed using generalised linear models. We used a binomial generalised linear model to define the effects of the host species, the date of discovery of carcasses, and the probability of an area to be positive for CDV. Moreover, the interaction at the first level, between the date and the probability of an area to be positive, was considered in order to evaluate the potential evolution of the outbreak. The statistical analyses were performed using the SPSS Statistics 20.01 software (IBM Corp., Armonk, NY, USA); values were significant when *p* < 0.05.

## 3. Results

### 3.1. Necropsy and Histopathological Analysis

Most of the carcasses found during the study were not suitable for an accurate necropsy and histopathological examination because of the advanced autolytic phenomena. Only a few red foxes were found in well-preserved conditions and subjected to a complete necroscopic examination. Severe weight loss, bilateral purulent conjunctivitis with ocular purulent discharge, and mucopurulent rhinorrhoea were frequently noted. The lung lesions consisted of multifocal diffused necrotic foci and severe pleuropneumonia, more commonly affecting the apices of the pulmonary lobes. Impaired gastrointestinal tract with severe catarrhal gastritis and enteritis, and a congested spleen and liver with severe necrotic-haemorrhagic pancreatitis were regularly recorded in all the examined carcasses. Other lesions observed in a few cases included plantar hyperkeratosis and bilateral nephrosis with focal haemorrhages in the cortex. A few cases of gastrointestinal nematode infestation and sarcoptic mange were also recorded. In several animals, traumatic lesions, probably due to vehicular collisions, were detected.

The scarcity of animals in well-preserved conditions prevented the application of common histopathological protocols, limiting the acquisition of useful information about the potential CDV infection. In the few cases that were histopathologically examined, the brain was found to be characterised by the cerebral sponginess of the white matter, also involving the cerebellum, strong gliosis, and presence of inflammatory lympho-plasmacellular infiltrates in the meninges. In the lungs, common interstitial pneumonia and strong congestion; characteristic eosinophilic intracytoplasmic and intranuclear inclusions in the bronchiolar and bronchial epithelium; and, in several cases, multifocal necrosis with the presence of pulmonary syncytial cells was observed. The lymph nodes and spleen showed hyperplasia, multifocal necrosis, lymphocyte depletion, and macrophage infiltration, along with the presence of multinucleated cells containing acidophilic inclusions. In particular, the spleen and liver were characterised by strong congestion; Kupffer cell hyperplasia and multifocal miliary necrosis were observed in the liver. Intense catarrhal inflammation was confirmed in the stomach and intestines. Eosinophilic intracytoplasmic inclusions were also occasionally observed in the bladder epithelium. Nephrosis characterised by the vacuolar degeneration of the epithelium of the urinary tubules, the multifocal necrosis of mesangial cells, and eosinophilic intracytoplasmic inclusions in the epithelial cells of the renal pelvis were observed.

### 3.2. Molecular and Phylogenetic Analyses

**Figure 2:**
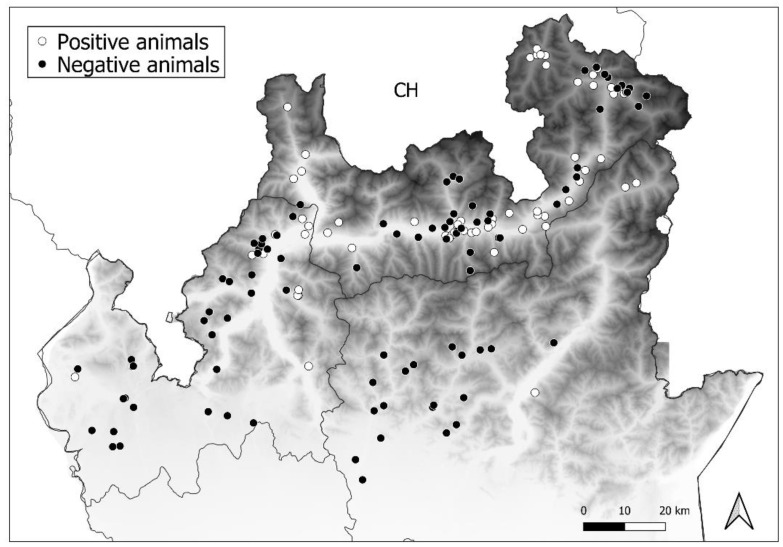
Distribution of positive (**white circles**) and negative animals (**black circles**) among the three study areas.

Nucleoprotein amplification allowed the detection of CDV in 50 foxes (*n* = 50/126, *p* = 39.7%, 95% confidence interval (CI): 31.6–48.4 exact Wilson), 10 badgers (*n* = 10/19, *p* = 52.6%, 95% (CI): 31.7–72.6), and 1 marten (*n* = 1/7, *p* = 14.3%, 95% [CI]: 2.6–51.3). Data on positive samples belonging to different areas are summarised in Table 2 and presented in Figure 2.

Subsequently, among the positive samples from the three areas, 40 foxes and all the positive badgers and martens were subjected to RT-PCR to amplify a portion of the H gene (sequence accession number: MW036774 to MW036824). This analysis did not allow the confirmation of positivity in samples derived from three foxes and a badger.

**Figure 3:**
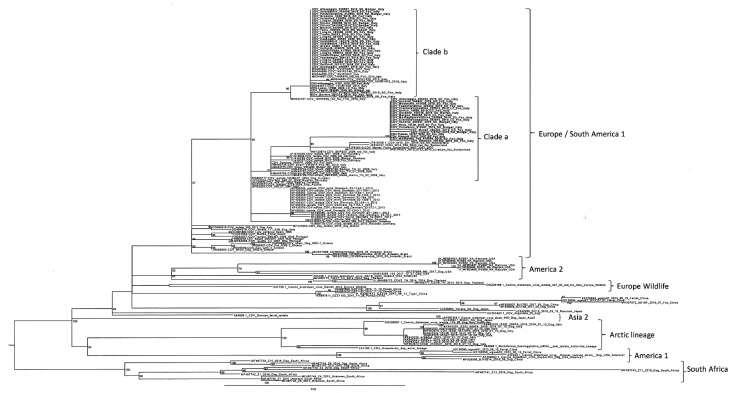
Maximum-likelihood phylogenetic tree based on the partial sequencing of the H gene. Italian CDV sequences investigated in this study are reported in bold. Reference CDV sequences are named, including the genBank accession number, the name of the strain, the host, and the country of origin. Boostrap values are indicated next to nodes.

Phylogenetic analyses based on partial sequencing of the H gene showed that our Italian CDV sequences belonged to the EU1 lineage and were categorised into two different clades. One clade (clade *a*) was closely related to the CDV sequences reported in the past epidemic wave of 2006–2010, which originated from wildlife in Germany and in the Alpine area covering the Italian, Swiss, and Austrian borders. The second clade (clade *b*) showed a close relationship, with the CDV sequences originating from the northeastern part of Italy (Veneto, Trentino, and South Tyrol) during the epidemic wave of 2011–2018 (Figure 3).

**Figure 4:**
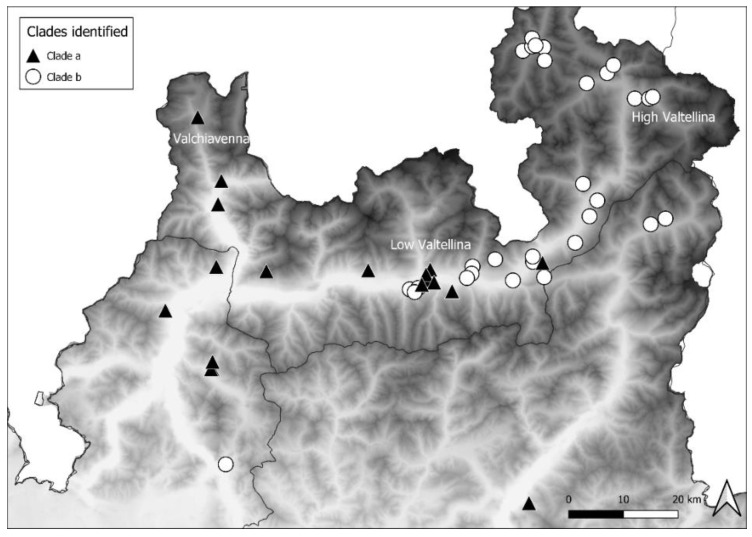
Distribution of clade *a* and *b* from Valchiavenna and High Valtellina, respectively.

The distribution of clade *a* mostly extended up to the western part of Area 3 and in Area 1, descending from Valchiavenna, and included 14 foxes, 3 badgers, and 1 marten. Clade *b* was mainly present in the eastern part of Area 3 and in Area 2, moving along High Valtellina, and involved 24 foxes and 6 badgers. Therefore, the distribution of the CDV sequences belonging to the two clades complied with the geographical conformation of the alpine valleys. In Area 3, near Low Valtellina, which connects the two above-mentioned valleys, the two different clades overlapped (Figure 4).

Sequencing data of the entire H gene of nine Italian CDV strains representative of clades *a* and *b* were obtained. A BLAST analysis of the nucleotide sequences showed that clade *a* had the highest percentage identity with the H04Bp1F strain isolated from a Hungarian dog in 2004, while that for clade *b* was with the SNP350/09 strain originating from a red fox in Italy in 2009 in an area corresponding to Area 3 of this study.

A molecular analysis of the deduced sequence of the protein H of the nine Italian CDV strains showed 98.2 to 98.8% identities with the W10/301F strain of the EU1 lineage. Of these, five sequences belonged to clade *a* and were highly homologous to each other (from 99% to 100% identity). The other four sequences, belonging to clade *b*, showed an identity of 99.7% to 99.8%. The amino acid substitutions observed in the consensus sequences of the Italian clades *a* and *b* were compared with the residues present in the homologous protein sequences of the most closely related strains of the EU1 lineage. Several amino acid substitutions were unique to the Italian strains and determined the two clades (Table 3).

Nine potential Asp-linked glycosylation sites (N-X-S/T) were predicted in residues 19–21, 149–151, 309–311, 391–393, 422–424, 456–458, 584–586, 587–589, and 603–605 of the H protein. Of these, six potential glycosylation sites were identified using the threshold value of 0.5 at positions 19, 149, 309, 391, 422, and 587, which were conserved across the Italian CDV strains and the related EU1 strains.

### 3.3. IEM Analysis

IEM analysis was performed on samples from 80 foxes, 16 badgers, and 7 martens. Positive samples showed immunoaggregates of viral particles or their fragments that could be morphologically attributed to CDV. In particular, a few broken particles with the typical external membrane showed short and regularly spaced tiny projections arranged in palisade, but more often large aggregates of the typical Paramyxovirus nucleocapsid chains were visible and were covered with a halo of antibodies. Among the samples analysed, 24 foxes were positive, and in particular the presence of CDV was confirmed by PCR in 22 samples. The remaining two cases were weakly positive for IEM analysis, and there was no concordance with PCR analyses. Among the badgers, five samples that were positive in the IEM analysis were also confirmed by the molecular methods. All the samples from martens were negative for IEM analysis. Moreover, a total of 15 foxes, 4 badgers, and 1 marten were positive for the molecular investigation but negative for IEM analysis.

### 3.4. Statistical Analyses

The probability of being infected was not influenced by the host species or the date of discovery of the carcass (*p* > 0.05) but was significantly influenced by the provenance area. Animals hailing from Area 3 had a higher probability of being infected than those from Areas 1 and 2 (*p* < 0.001), and no differences between Areas 1 and 2 were recorded. The interaction between the date of discovery of the carcass and provenance area was not statistically significant (*p* > 0.05).

## 4. Discussion

The present study highlighted the circulation of CDV in the northern part of Lombardy (Italy), with a percentage of positive animals of 39.7% in foxes, 50% in badgers, and 14.3% in stone martens, confirming the heterogeneous susceptibility of all the three species investigated. Mustelids are one of the species most susceptible to CD [2]. Furthermore, higher prevalence is usually registered in stone martens, since it is considered the most synanthropic species [12,21]. However, our data only partially support the findings of previous studies, since, in proportion, a higher number of positive animals among the badgers was recorded. With regard to stone martens, only one positive for CDV infection was detected, but a limited number of carcasses was found, probably because of their smaller size and elusive behaviour, which makes sampling difficult. We did not find any carcass of other species of mustelids. However, sampling based on passive surveillance can be influenced by several factors such as the season and viability of the environment, as well as the presence and abundance of specific species. Thus, some data on disease prevalence in wild species may be lost. Nonetheless, despite clear sampling limits, statistical analyses showed that the host species did not have a significant effect on the probability of CDV infection.

The three target species are representative of pre-alpine and alpine environments. However, most animals usually live on the valley floor at lower altitudes, where human presence and activity are mainly present. Statistical analyses showed that the animals sampled in Area 3 had a higher probability of being infected than animals from Areas 1 and 2. In Area 3, which is mostly characterised by pre-alpine and alpine environments, the population density of foxes seems to be higher than that in Areas 1 and 2. Census data on foxes are currently unavailable, since there are no evaluation systems in place. Therefore, the indication of a high density in Area 3 represents relative information based on the frequencies of observation and human contact with foxes. Regardless of the ecological characteristics of their habitat, in the past decades the increase in ungulates, above all in the Alps, has likely favoured the presence of medium-sized carnivores such as foxes, and thus the increase in host density may have strengthened the virus transmission efficacy [10]. Moreover, in this area, there is intense human activity, in terms of industries and the presence of numerous agro-pastoral realities, including vital viticulture, and this represents a popular tourist destination throughout the year. Frölich et al. [21] assumed a positive correlation between the human population density and the density of domestic dogs, which is considered as a contamination source of the habitat of free-ranging carnivores. Indeed, in several previous studies and the phylogenetic analyses of the present study, a relationship between sequences of dog CDV strains and strains identified in wild foxes was recorded [14,21,22,23]. This suggests horizontal transmission between dogs and wild carnivores and among wild carnivores as well. Thus, dogs represent the reservoir of CDV for wild carnivores, and the frequency of infection in wildlife could be directly proportional to the number of infected dogs. However, this eventuality mainly concerns the EU3 lineage in the southern Italy, while in Lombardy this does not likely represent a source of diffusion, considering the high level of vaccination in pets and the lack of detection of CDV in domestic dogs.

Phylogenetic analyses showed the presence of two different clades, which have previously been described as corresponding to two different epidemic waves reported in 2006–2009 and 2011–2018, respectively [24]. It is interesting to note that our study refers to only a single period, the epidemic wave of 2018–2020, and therefore, the distribution of our CDV sequences in two clades is not related to the different times but to the different geographical origins. In particular, sequences isolated in Valchiavenna (northwestern part of Lombardy) were placed in clade *a*, closely related to sequences recognised during the Swiss and Bavarian outbreaks that occurred in 2007–2009. Since the first cases reported were near the boundary of the country, a potential displacement of infected foxes from Switzerland could be hypothesised. Indeed, the sequences of strains isolated from stone martens, foxes, and lynxes detected in that country clustered together with the sequences of the strains in the present study.

Foxes likely play a major role in CDV spread due to their wide geographic range and social behaviour during the reproductive season and the dispersion of juvenile animals [25]. Alternatively, a long-term persistence of CDV in wildlife could occur for decades, sometimes not being associated with fatal consequences [12]. Animals with robust immune responses can recover by developing life-long immunity to reinfection [9]. These aspects could explain the long periods of no evidence of cases in Italy and central Europe [12,25]. The emergence of new outbreaks could suggest a slight increase in CDV pathogenicity, as observed in the Swiss cases characterised by accentuated neurotropism [12]. Hence, it is possible that wild carnivores are no longer immunocompetent, leading to new CDV epidemics. Unfortunately, in the present study, blood samples were not available in order to conduct a serological investigation on wild carnivores. In fact, the majority of the animals were found dead; in some cases, they were likely victims of road accidents; thus, collection of blood and evaluation of any neurological symptoms were very difficult.

The second clade (clade *b*) observed is represented by sequences closely related to the “second wave” of CDV that occurred from 2011 to 2018, and was likely introduced from the Balkans [24]. Unlike the previous outbreaks in the northeastern part of Italy (Veneto, Trentino, and South Tyrol), which moved southward, this epidemic wave seemed to spread westward, particularly in Northern Lombardy. We analysed potential space-time evolutions, but statistical analyses did not highlight any correspondence between territory and temporal case progression. However, it was possible to observe a progressive spread of the two clades along the valleys up to the middle of Low Valtellina, where their distribution overlapped. Moreover, the last cases recorded in 2020 were located further in the south compared with the previously recognised cases.

The presence of sequences related to strains identified in previous outbreaks could suggest the stability of the infection within wild carnivore populations, and the distribution of infection is likely related to the movement of foxes along the valleys, whose topographical conformation could favour the maintenance of CDV by inducing foxes to concentrate in determined areas. Moreover, the disease probably does not reach a mortality rate high enough to decrease the foxes’ densities, allowing new susceptible hosts to be infected through natal recruitment.

Considering the results of necropsy and histopathological analyses, classical signs and microscopic lesions were detected in most cases. Only a few cases could be compared with those caused by the Swiss strains, which were characterised by accentuated neurotropism [12], particularly with the detection of demyelinating lesions involving the cerebellum. However, we found intranuclear or intracytoplasmic eosinophilic inclusions only in glial cells but not in neurons. Furthermore, we did not observe polioencephalitis, but common demyelinating lesions were observed. Nonetheless, it would have been appropriate to increase the number of cases analysed by histopathology in order to validate the potential accentuated neurotropism, but limits related to the sampling prevented the collection of a suitable number of samples. In this regard, the systematic application of PCR protocols for CDV identification allowed the discovery of the presence of the virus in animals whose clinical signs or symptoms were ignored or in carcasses showing advanced autolytic phenomena, which is not adequate for anatomo-pathological examinations. Likewise, the use of diagnostic tools, such as IEM, allows obtaining valuable information about the presence and severity of viral infections. Even though it is considered less sensitive than PCR, for monitoring wildlife it represents a good screening method in the absence of anamnesis during passive surveillance plans, since it can detect both acute phases of CDV infection, giving additional information to the qualitative results of PCR, and the presence of any other co-infecting agent eventually present in the examined organs.

In conclusion, this study describes the evolution of CDV infection occurring in wild carnivores from 2018 in Lombardy, Italy, highlighting the emergence of two contemporary outbreaks related to the two previous epidemic waves in northeastern Italy [24]. The evidence of the presence of sequences related to the strains involved in the outbreaks that occurred in 2008 supports the hypothesis that CDV is well adapted to wild carnivores, mostly circulating with sub-clinical manifestations and cyclically leading to mortality in the fox population as well as in other susceptible species. However, CDV does not seem to have a severe impact on this population; thus, their potential role of being a reservoir for the endangered species highlights their importance. Therefore, the early detection of CDV in wildlife species is of considerable importance, and the application of molecular analyses to examine the organs of wild animals, even with lacking clinical history or in the absence of specific lesions, allows the detection and distribution of viruses in relation to the different and new circulating genotypes.

## Figures and Tables

**Table 1:** Italian CDV strains representatives of clades *a* and *b*, for which the complete H gene is given.

Clade	Strain	Acc Number H Complete
*b*	CDV_Chiuro_250358_2019_SO_Badger_Italy	MW036782
*b*	CDV_Temu_306003_2018_BS_Badger_Italy	MW036808
*b*	CDV_VillaDiTirano_396501_2019_SO_Fox_Italy	MW036820
*b*	CDV_Valtellina_208435_2018_SO_Fox_Italy	MW036817
*a*	CDV_Berbeno_181902_2019_SO_Fox_Italy	MW036777
*a*	CDV_Modesimo_410638_2018_SO_Fox_Italy	MW036798
*a*	CDV_Gordone_193148_2019_SO_Marten_Italy	MW036787
*a*	CDV_Chiavenna_312067_2019_SO_Badger_Italy	MW036781
*a*	CDV_Musso_282223_2019_CO_Fox_Italy	MW036800

**Table 2:** Number of animals collected from the three areas and related positive samples (Pos: positive).

AREA	FOX			BADGER			MARTEN		
	Nr	Pos	P(%) CI95%	Nr	Pos	P(%) CI95%	Nr	Pos	P(%) CI95%
1	41	11	26.8% (15.7–41.9)	3	1	33.3% (6.1–79.2)	1	0	0%
2	20	2	10.0% (2.8–30.1)	2	1	50.0% (9.4–90.5)	-	-	-
3	65	37	56.9% (44.8–68.2)	14	8	57.1% (32.5–78.6)	6	1	16.7% (3–56.3)
TOT	126	50	39.7% (31.6–48.4)	19	10	52.6% (31.7–72.6)	7	1	14.3% (2.6–51.3)

**Table 3:** Amino acid substitutions across the consensus sequences of the Italian clades *a* and *b* and their closely related strains of the EU1 lineage. Amino acid mutations defining clades *a* and *b* are identified in bold (H: hemagglutinin).

H Protein	W10/301F	H04Bp1F	SNP350/09	BV4	Clade *a*	Clade *b*
Position						
71	N	S	S	S	S	S
129	R	R	R	R	**K**	R
159	V	I	I	I	I	I
174	A	A	A	A	**T**	A
178	G	G	G	G	G	**S**
195	M	V	M	V	M	V
198	V	V	V	V	**F**	V
238	Y	Y	Y	Y	Y	**F**
246	Q	Q	Q	Q	**E**	Q
273	V	V	I	V	V	V
278	S	S	S	S	**P**	S
301	T	T	T	T	N/V	N
365	A	V	A	A	A	A
423	V	I	V	V	V	I
500	M	M	M	L	M	M
517	N	N	N	N	N	**S**
538	V	V	V	V	V	**I**
542	I	I	I	I	I/S	I
549	H	Y	H	H	H	Y
580	R	R	R	Q	R	R

## Data Availability

Data generated or analysed during this study are included in the published article. RNA sequencing data were deposited in GenBank, Accession Number MW036774 to MW036824.
